# Measuring Caribbean stress and resilient coping: Psychometric properties of the PSS-10 and BRCS in a multi-country study during the COVID-19 pandemic

**DOI:** 10.1017/gmh.2024.83

**Published:** 2024-10-11

**Authors:** Michael H. Campbell, Jill Gromer-Thomas, Katija Khan, Bidyadhar Sa, Paula M. Lashley, Damian Cohall, Christine E. Chin, Russell B. Pierre, Nkemcho Ojeh, Ambadasu Bharatha, Heather Harewood, O. Peter Adams, Md. Anwarul Azim Majumder

**Affiliations:** 1 Faculty of Medical Sciences, The University of the West Indies, Cave Hill Campus, Barbados; 2 College of Social Work, Florida State University, Tallahassee, FL, USA; 3 Faculty of Social Sciences, The University of the West Indies, St. Augustine Campus, Trinidad & Tobago; 4 School of Clinical Medicine and Research, The University of the West Indies, Nassau Campus, The Bahamas; 5 Faculty of Medical Sciences, The University of the West Indies, Mona Campus, Kingston, Jamaica

**Keywords:** stress, resilience, coping, Caribbean, confirmatory factor analysis

## Abstract

Caribbean health research has overwhelmingly employed measures developed elsewhere and rarely includes evaluation of psychometric properties. Established measures are important for research and practice. Particularly, measures of stress and coping are needed. Stressors experienced by Caribbean people are multifactorial, as emerging climate threats interact with existing complex and vulnerable socioeconomic environments. In the early COVID-19 pandemic, our team developed an online survey to assess the well-being of health professions students across university campuses in four Caribbean countries. This survey included the Perceived Stress Scale, 10-item version (PSS-10) and the Brief Resilient Coping Scale (BRCS). The participants were 1,519 health professions students (1,144 females, 372 males). We evaluated the psychometric qualities of the measures, including internal consistency, concurrent validity by correlating both measures, and configural invariance using confirmatory factor analysis (CFA). Both scales had good internal consistency, with omega values of 0.91 for the PSS-10 and 0.81 for the BRCS. CFA suggested a two-factor structure of the PSS-10 and unidimensional structure of the BRCS. These findings support further use of these measures in Caribbean populations. However, the sampling strategy limits generalizability. Further research evaluating these and other measures in the Caribbean is desirable.

## Impact statement

This study addresses the need for culturally relevant and empirically validated measurement tools in mental health research in the Caribbean. Traditionally, the region has depended on psychological measures developed elsewhere, usually in North America or Europe, and relatively few have established psychometric properties for Caribbean populations. By providing support for the use of the Perceived Stress Scale (PSS-10) and Brief Resilient Coping Scale (BRCS) in the Caribbean setting, the current study enables more contextualized and culturally responsive measurement of key indicators of health and wellbeing. Research using these measures is useful to understand the complex dynamics of stress in the Caribbean, including factors such as climate threats, economic challenges, and social and political disruptions, which have been particularly pronounced during the COVID-19 pandemic. A more comprehensive understanding is crucial for creating mental health interventions and policies that address the needs of Caribbean people. These measures have broad application to support the work of health researchers, healthcare providers, policymakers and educational institutions in the region. The focus on health professions students is especially relevant, given their future role in promoting individual and community wellbeing in a region beset with vulnerabilities. More immediately, this study can inform efforts to develop effective mental health resources in Caribbean settings. This research further contributes to the broader conversation on global mental health, underscoring the importance of measures that are culturally valid. The study may be a useful example for other regions with similar needs for tailoring mental health tools to local contexts. In summary, this paper advances research on stress and coping in the Caribbean and contributes to the growing body of culturally responsive measures in global mental health.

## Introduction

Measures with established reliability and validity in the English-speaking Caribbean are key for mental health research and practice. Efforts to develop a body of instruments for appropriate use involve both development of novel scales and psychometric evaluation in the Caribbean of those developed elsewhere (Alea and Ali, 2018). Existing Caribbean mental health research has overwhelmingly employed measures developed and normed outside of the region, primarily in North America or Europe, and has only rarely evaluated the psychometric properties or contextual use of these instruments; and when doing so, studies have infrequently moved beyond cursory reports of Cronbach’s alpha (Lambert et al., 2016, 2018).

As the need for understanding climate threat in small island developing states (SIDS) increases, the regional literature on climate and health has not kept pace, and considerable gaps in knowledge of Caribbean climate threats, including mental health sequelae, persist (Rise et al., 2022). These gaps are crucial in a region with complex and dynamic vulnerabilities. Stressors experienced by Caribbean people are longstanding and multifactorial, as emerging climate threats are layered onto existing geographic, economic, social, political and health systems, whose dynamics vary among countries in the region (Granger, 1997). Moreover, climate change may threaten post-colonial gains and needed further steps for sustainable development (Rhiney and Baptiste, 2019).

Regional mental health professionals have responded to climate threats relatively quickly in terms of providing clinical services and policy consultation (e.g., Shultz et al., 2020; Torres-Llenza and Safran, 2021), and research efforts are now emerging (e.g., Seon et al., 2024), with several studies still in development or in press (Campbell and Greaves, 2022). For example, further study is needed on the psychological and behavioral implications of rising sea levels, ocean acidification and heat stress (Birthwright and Smith, 2023). Relevant and psychometrically sound measures are essential to conduct this research and for the broader study and practice of public mental health. Notably, Caribbean practitioners have identified barriers posed by lack of appropriate clinical tools for use in disaster mental health interventions (Dudley-Grant and Etheridge, 2007).

Against this background, the COVID-19 pandemic presented an acute threat that has resulted in negative mental health outcomes, notably anxiety and depression, globally. These symptoms generally increased in concert with restrictions on activity, but there has been considerable variability across settings and populations (Salanti et al., 2022). Disruptions in medical and health professions (MHP) education were widespread, and there has been a proliferation of studies examining the impact of COVID-19 on the well-being of MHP students. A recent meta-analysis of 201 studies including 198,000 medical students (Peng et al., 2023) identified pooled prevalences for stress (34%), anxiety (38%), depression (41%), sleep disorder (52%), general psychological distress (58%), post-traumatic stress disorder (34%), suicidal ideation (15%) and burnout (36%). Studies across allied health professions have shown broadly consistent patterns of these symptoms (Chutiyami et al., 2022; Pfeifer et al., 2022).

Our research team was tasked in early 2020 with developing a rapid response survey to assess the well-being and academic performance of health professions students across The University of the West Indies campuses in the Caribbean, affording the opportunity to collect psychometric data from young adults in several Caribbean countries: The Bahamas, Barbados, Jamaica and Trinidad and Tobago. This coincided with emergency adaptations implemented by the university in response to the pandemic, including abrupt transition to blended and online learning (Cockburn and Chami, 2022) and comprehensive efforts to provide research and clinical support to Caribbean countries (Landis, 2021). Therefore, an important goal of the study was to inform efforts to provide student support in the early stages of the pandemic, when significant knowledge gaps hindered planning. The study further presented an opportunity to establish the psychometric properties of measures included in the survey with a large regional Caribbean sample.

We elected to include the Perceived Stress Scale, 10-item version (PSS-10; Cohen and Williamson, 1988) and Brief Resilient Coping Scale (BRCS; Sinclair and Wallston, 2004) to assess stress and coping behavior among MHP students. The PSS-10 is the most widely used self-report measure of stress in health research with substantial psychometric evidence supporting its use globally (Crosswell and Lockwood, 2020; Kogar and Kogar, 2023). Additionally, there is considerable precedent for using the PSS-10 in research with MHP students, including a number of studies in the COVID-19 era (e.g., Puranachaikere et al., 2021; Twardowski et al., 2023; Williams et al., 2020). Although the BRCS was developed more recently, the instrument has similarly been utilized in studies of coping behavior of medical and health professions students (Button et al., 2023; Heinen et al., 2017). Both measures are available for use without proprietary fees.

The PSS-10 measures the extent to which respondents appraise their current life circumstances as stressful. The instrument seeks to “tap how predictable, uncontrollable and overloaded respondents find their lives” (Cohen and Williamson, 1988, pp. 33–34). The instrument is a briefer measure derived from the original 14-item scale (Cohen et al., 1983). The PSS-10 has demonstrated strong psychometric properties, including configural invariance, in a variety of settings and age groups (Lee, 2012). Most studies using structural equation modeling (SEM) have reported a two-factor structure comprising perceived helplessness (negatively worded items: 1, 2, 3, 6, 9, 10) and perceived self-efficacy (positively worded items: 4, 5, 7, 8) (Yılmaz Koğar and Koğar, 2024). The PSS-10 has been widely validated and employed for research in LMICs and, therefore, is an important tool for global mental health research (see, e.g., Adamson et al., 2020; Katus et al., 2022; Lai et al., 2020). A previous SEM study in Barbados (Campbell et al., 2019) found acceptable fit for a two-factor model of the PSS-10. However, further analysis of this data indicated a better fit and improved internal consistency for a revised 7-item scale.

The BRCS is a very brief (4-item) scale designed to measure adaptive coping behaviors in response to stress. The instrument was originally developed for use with patients with rheumatoid arthritis (Sinclair and Wallston, 2004), but subsequent studies have established validity and a unidimensional factor structure in more general populations, including a nationally representative sample in Germany (Kocalevent et al., 2017) and university students in Spain (Limonero et al., 2014). The BRCS has been widely used in recent studies of coping among healthcare providers (HCPs) during the COVID-19 pandemic, including in Egypt (Khalaf et al., 2020; Sehsah et al., 2021) and Ethiopia (Tsehay et al., 2020). Cheng et al. (2022) conducted a meta-analysis of resilience among HCPs during the pandemic using several measures, including the BRCS. Some BRCS studies during the pandemic assessed configural invariance and generally supported a unidimensional factor structure, for example, in Italy (Murphy et al., 2021) and Thailand (Nochaiwong et al., 2022).

The current study, with a larger and broader regional sample than our previous paper (Campbell et al., 2019), seeks to evaluate the appropriateness of the PSS-10 and BRCS for future Caribbean research and to provide a more robust assessment of the psychometric strengths and configural invariance of the measures. Therefore, the aims of this paper are: 1) to describe the presentation of stress and coping behavior reported by Caribbean MHP students in the early stages of the COVID-19 pandemic; and 2) to evaluate the internal consistency, construct validity and factorial structure of the PSS-10 and BRCS in a sample including four Caribbean countries.

## Methods

### Study design and study sample

This paper capitalizes on a larger, emergently designed cross-sectional study meant to assess the unique needs of MHP students during the early to middle phases of the COVID-19 pandemic. We conducted an anonymous online survey using voluntary response sampling via SurveyMonkey to explore the relationships among readiness for online learning, stress and coping among undergraduate MHP students at The University of the West Indies campuses in The Bahamas, Barbados, Jamaica and Trinidad and Tobago. All MHP students currently enrolled during April–June 2020, during the emergency transition to virtual learning in the early phase of the COVID-19 pandemic, were invited to participate.

### Measures

- Sociodemographic Questionnaire: Participants were asked to provide information on their age, gender, campus country and academic program.
- Perceived Stress Scale (PSS): The PSS-10 (Cohen and Williamson, 1988) was used to assess participants’ perceived stress levels. This 10-item scale measures the degree to which situations in one’s life are appraised as stressful, with higher scores indicating higher levels of perceived stress. Possible scores range from 0 to 40. Although formal cut-off scores are not established, interpretive ranges are 0–13 (minimal stress), 14–26 (moderate stress) and 27–40 (high stress).
- Brief Resilient Coping Scale (BRCS): The BRCS (Sinclair and Wallston, 2004) was used to assess participants’ ability to cope with stress in a resilient manner. This 4-item scale measures the ability to bounce back or recover from stress, with higher scores indicating greater resilience. Possible scores range from 4 to 20. Interpretive ranges are 4–13 (low resilient coping), 14–16 (medium resilient coping) and 17–20 (high resilient coping).

### Ethical considerations

The study protocol was approved by the following research ethics committees: (i) the University of the West Indies-Cave Hill/Barbados Ministry of Health Research Ethics Committee/Institutional Review Board, Barbados (IRB No. 200403-B; April 27, 2020) and (ii) the Campus Research Ethics Committee, the University of the West Indies, St Augustine Campus, Trinidad and Tobago (IRB No. 200403-B; April 28, 2020). No identifying information was collected from participants. The research protocol complied with the Declaration of Helsinki.

### Statistical analysis

We examined the psychometric properties and factorial structure of the PSS-10 and BRCS. We evaluated the internal consistency of each measure using both Cronbach’s alpha (Cronbach, 1951) and McDonald’s Omega (McDonald, 1986). Then, concurrent validity was assessed by correlating both measures. Finally, we explored configural invariance using confirmatory factor analysis (CFA) to test the two-factor structure of the PSS-10 and unidimensional structure of the BRCS reported in previous research. Analyses were conducted with SPSS Version 29.0 and Mplus 6.

## Results

### Participants

The sample size for the overall study was 1,519 undergraduate MHP students (1,144 females, 372 males, 3 other/did not say). The distribution of participants by campus country is shown in Table 1. The respondents included 1,122 medical (MBBS) students (73.9%); the next largest group comprised 113 pharmacy students (7.4%), followed by 66 dentistry students (4.3%). The remaining students were enrolled in a variety of allied health programs (Table 1). The average age of respondents was 22.9 years (SD = 3.57; range: 18–48).

**Table 1.** Gender, program and campus country of participants

Background information	Respondents (%)
Gender (*n* = 1,514)	
Female	1,139 (75.4%)
Male	372 (24.6%)
Other/no response	3
Program (*n* = 1,512)	
MBBS	1,119 (74%)
Pharmacy	113 (7.5%)
Dentistry	66 (4.4%)
Bachelor of health sciences	37 (2.5%)
Optometry	34 (2.3%)
Nursing	33 (2.2%)
Veterinary medicine	30 (2%)
Physical therapy	27 (1.8%)
Basic medical sciences	20 (1.3%)
Diagnostic imaging	18 (1.2%)
Other	15 (1%)
Campus (*n* = 1,513)	
St Augustine (Trinidad & Tobago)	606 (40.1%)
Mona (Jamaica)	494 (32.7%)
Cave Hill (Barbados)	339 (22.4%)
Nassau (The Bahamas)	74 (4.9%)

### Initial data screening

Of the 1,519 participants, 1,420 and 1,437 completed the PSS-10 and BRCS, respectively, and were included in the psychometric analysis. No more than 0.3% of data were missing for any item of the PSS-10 or BRCS, and Little’s (1988) test suggested that data were missing completely at random for both the PSS-10 (*χ* ^2^ (36) = 50.01, *p* = 0.06) and BRCS (*χ* ^2^ (13) = 18.91, *p* = 0.15). Given this, estimation maximization (EM) was used to impute missing data.

### Summary of scores, reliability and validity of the PSS-10 and BRCS

Scores on the PSS-10 ranged from 0 to 40, with a mean of 24.16 (SD = 7.70). Scores on the BCRS ranged from 4 to 20, with a mean of 13.33 (SD = 3.39).

Given concerns about the restrictive assumptions of Cronbach’s alpha (McDonald, 1986) and recommendations of Dunn et al. (2014), we calculated both alpha and McDonald’s omega to assess the internal consistency of both measures (and have reported these to three decimal places to illustrate very modest differences). Internal consistency for the PSS-10 (*α* = 0.910; *ω* = 0.912) was excellent according to both measures, which were essentially equivalent in this case. Internal consistency for the PSS-10 subscales was acceptable for perceived self-efficacy (*α* = 786; *ω* = 0.786) and good for perceived helplessness (*α* = 0.897; *ω* = 0.897). All items contributed to the full scale and their respective subscales.

Internal consistency for the BRCS was good (*α* = 0.810; *ω* = 0.813) but less robust than for the PSS-10. Differences between methods of calculating internal consistency were minimal. All items contributed to the full BRCS scale.

Concurrent validity of the PSS-10 and BRCS were examined using Pearson correlation (BRCS; *n* = 1,420, *r* = −0.44, *p* < 0.01). The observed moderate negative correlation between the two scales was as expected.

### Factor structure

**Figure 1.**
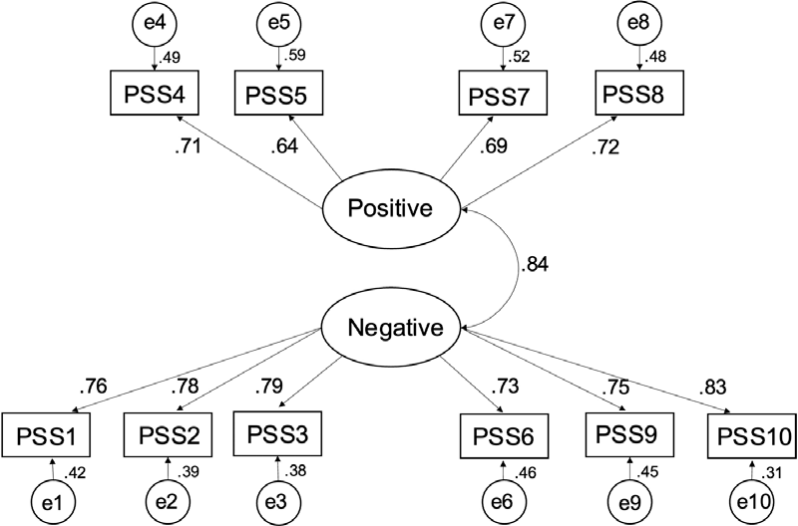
Path diagram of two-factor model of Perceived Stress Scale (PSS-10).

PSS-10. A CFA using maximum likelihood estimation examined the fit of the two-factor model reported in other samples. Alternate models, including a second-order model and a model of the abbreviated 7-item scale, were also tested but found to have inadequate fit to the data. All items were related to their associated factors, with factor loadings ranging from 0.64 (item 5) to 0.83 (item 10). The path diagram of this model is shown in Figure 1. Except for chi-square and relative chi-square, the fit indices (Table 2) calculated by Mplus 6 were within the acceptable ranges outlined by Kline (2016). The chi-square statistic and its transformations are not generally reliable as bases for model acceptance due in part to sensitivity to sample size (Schermelleh-Engel et al., 2003; Vandenberg, 2006). Further, while the fit indices are acceptable under Kline’s (2016) rubric, there are divergent perspectives on model fit (e.g., Hu and Bentler, 1999; Marsh et al., 2004) and under some other standards, the RMSEA would be considered moderate or mediocre.

**Table 2.** Fit indices for Perceived Stress Scale (PSS-10) CFA model

	*χ* ^2^	df	*χ* ^2^ *p* value	*χ* ^2^/*df*	CFI	TLI	SRMR	RMSEA	RMSEA 90% C.I.
Two–factor, 10–item	327.72	34	<0.05	9.64	0.96	0.95	0.028	0.078	0.070–0.086
Acceptable Range	N/A	N/A	*p* ≥ 0.05	<3	≥0.90	≥0.95	<0.08	<0.08	N/A

*Note.* CFI = comparative fit index; TLI = Tucker-Lewis index; SRMR = standardized root mean square residual; RMSEA = root mean square error of approximation; C.I. = confidence interval.

*Note.* Acceptable ranges sourced from Kline (2016).

**Figure 2.**
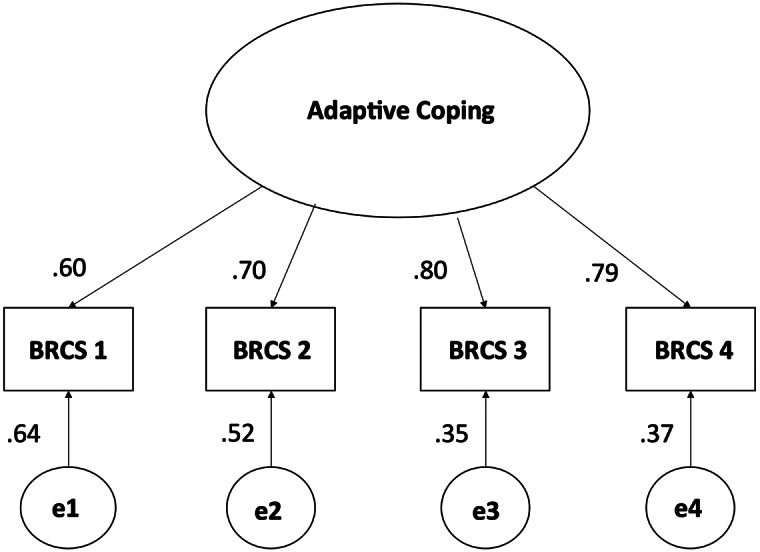
Path diagram of one-factor model of Brief Resilient Coping Scale (BRCS).

BRCS. A CFA using maximum likelihood estimation confirmed the unidimensional structure of the BRCS. A second-order model was also tested but found to have inadequate fit to the data. Factor loadings were acceptable, ranging from 0.60 (item 1) to 0.80 (item 3). The path diagram for this model is shown in Figure 2. Fit indices, which, except for chi-square and relative chi-square, were all in acceptable ranges, are shown in Table 3. Again, the RMSEA meets the standards used in this study (Kline, 2016) but may be considered moderate by those holding diverging perspectives (Hu and Bentler, 1999; Marsh et al., 2004).

**Table 3.** Fit indices for Brief Resilient Coping Scale (BRCS) CFA model

	*χ* ^2^	df	*χ* ^2^ *p* value	χ^2^/*df*	CFI	TLI	SRMR	RMSEA	RMSEA 90% C.I.
One–factor	15.83	2	<0.01	7.92	0.99	0.98	0.014	0.069	0.040–0.103
Acceptable range	N/A	N/A	*p* ≥ 0.05	<3	≥0.90	≥0.95	<0.08	<0.08	N/A

*Note.* CFI = comparative fit index; TLI = Tucker-Lewis index; SRMR = standardized root mean square residual; RMSEA = root mean square error of approximation; C.I. = confidence interval.

*Note.* Acceptable ranges sourced from Kline (2016).

Second-order models for both scales were tested for both the PSS-10 and BRCS, but the fit was not adequate for either measure using criteria from Kline (2016).

### Gender differences

Significant differences in PSS-10 (*t*(1405) = −4.59, *p* < 0.01) and BRCS scores (*t*(1418) = 3.76, *p* < 0.01) by gender were observed using independent *t* tests. For men (*n* = 338), the mean PSS-10 score was 22.49 (SD = 8.08). For women (*n* = 1,069), the mean PSS-10 score was 24.68 (SD = 7.50). For men (*n* = 344), the mean BRCS score was 13.93 (SD = 3.30). For women (*n* = 1,084), the mean BRCS score was 13.14 (SD = 3.40).

## Discussion

Findings support a two-factor structure of the PSS-10 comprising perceived helplessness and perceived self-efficacy subscales and provide further evidence of configural invariance, which is key for comparative research. Internal consistency was acceptable for the PSS-10 and its component subscales. Further, findings support a unidimensional structure of the BRCS and show adequate internal consistency for this measure of resilient coping, at least among the study population of future medical and health professionals in the Anglophone Caribbean. This is evidenced by the fit indices, aside from chi-square and relative chi-square, which are often reported but also outdated and problematic (Schermelleh-Engel et al., 2003; Vandenberg, 2006). Although these indices were within the acceptable ranges outlined by Kline (2016) for both scales, there are divergent perspectives on model fit (e.g., Hu and Bentler, 1999; Marsh et al., 2004) and it should be mentioned as a caveat that under some other standards, the RMSEA for both scales would be considered a moderate and therefore questionably acceptable fit.

Even so, the pattern of findings is consistent with extant literature (Kogar and Kogar, 2023), including other Caribbean samples (Campbell et al., 2019), so we believe that our data provide support for the valid use of the PSS and BRCS among Caribbean healthcare students.

Specifically, these results provide initial support for use of the BRCS as well as increased support for use of the PSS-10 in the Caribbean, building on a previous study (Campbell et al., 2019), which included only Barbados and suggested that a modified 7-item scale would provide better fit. Importantly, the two-factor model reported in the current study included all 10 items of the measure, establishing support for use of the unmodified original PSS-10 (Cohen and Williamson, 1988) in Caribbean settings.

This study has several important limitations. Voluntary sampling, restriction of participants to MHP students and data collection during the COVID-19 timeframe are important limitations to generalizability of findings. Further research evaluating and employing measures of stress and coping in broader Caribbean contexts is desirable. Several other limitations of this study can be addressed in future research. Because this paper capitalizes on a larger, emergently designed study meant to assess the unique needs of health professions students during the COVID-19 pandemic, some important features of a typical psychometric validation study are absent. For example, test–retest reliability and social desirability among participants were not examined.

With these limitations acknowledged, this paper makes an important contribution by examining the psychometric properties of the PSS-10 and BRCS in a large multi-country sample. Valid and reliable measures of stress and coping are especially important for people living in vulnerable settings, including SIDS, where existing socioeconomic stressors are now compounded by increasing climate threats to health and wellbeing. Measures for these important constructs lack empirical support for use in the Caribbean, where few measures for clinical or research have documented psychometric properties.

In addition to evaluating the appropriateness of the PSS-10 and BRCS for use in the Caribbean, this study sought to describe the presentation of stress and coping behavior reported by Caribbean MHP students in the early stages of the COVID-19 pandemic. Women in this study reported significantly more perceived stress than men, and perceived stress was negatively correlated with resilient coping scores. Extant literature indicates that North American and European women experience more stressful life events and have higher levels of perceived stress (e.g., Costa et al., 2021). Globally, however, the effect sizes of gender differences in perceived stress and burnout vary by both region and occupation, suggesting that these differences are highly contextualized (Purvanova and Muros, 2010; Templeton et al., 2019). It is worth examining whether the gender differences found here exist outside of the pandemic crisis and whether this difference varies with profession or field of study. Further work should first focus on examining whether the observed gender differences persist beyond the acute phase of the pandemic. Thereafter, research to identify and address factors in Caribbean medical education that contribute to these differences is needed.

## Conclusion

Establishing acceptable psychometric properties of the PSS-10 and BRCS in the Caribbean context provides a useful contribution to the armamentarium of measures for Caribbean mental health research. Robust tools for assessing stress and coping are especially important in SIDS, whose people face a complex array of vulnerabilities related to geography, climate threat and socioeconomic factors. Further, both measures are tools for culturally responsive mental health research, intervention and policy development. Future research should further establish use of these scales across diverse communities and contexts in the Caribbean and other SIDS. Established measures of stress and coping are crucial for further work supporting regional resilience efforts in the context of socioeconomic and climate-related stressors.

## Data Availability

The data that support the findings of this study are available from the corresponding author upon reasonable request.
