# Supplementary material for: Measuring Caribbean stress and resilient coping: Psychometric properties of the PSS-10 and BRCS in a multi-country study during the COVID-19 pandemic
Source: Glob Ment Health (Camb). 2024 Oct 11;11:e77. doi: 10.1017/gmh.2024.83 (PMC11505040; doi:10.1017/gmh.2024.83)
Supplement: Peer review history

# Editor report: Recommendation: Measuring Caribbean stress and resilient coping: Psychometric properties of the PSS-10 and BRCS in a multi-country study during the COVID-19 pandemic — R0/PR2

**DOI:** 10.1017/gmh.2024.83.pr2

**Affiliations:** Primary Healthcare Sciences, University of Zimbabwe Faculty of Medicine, Zimbabwe

No accompanying comment.

# Editor report: Decision: Measuring Caribbean stress and resilient coping: Psychometric properties of the PSS-10 and BRCS in a multi-country study during the COVID-19 pandemic — R0/PR3

**DOI:** 10.1017/gmh.2024.83.pr3

**Affiliations:** Johns Hopkins University Bloomberg School of Public Health, United States

No accompanying comment.

# Editor report: Recommendation: Measuring Caribbean stress and resilient coping: Psychometric properties of the PSS-10 and BRCS in a multi-country study during the COVID-19 pandemic — R1/PR5

**DOI:** 10.1017/gmh.2024.83.pr5

**Affiliations:** Primary Healthcare Sciences, University of Zimbabwe Faculty of Medicine, Zimbabwe

No accompanying comment.

# Editor report: Decision: Measuring Caribbean stress and resilient coping: Psychometric properties of the PSS-10 and BRCS in a multi-country study during the COVID-19 pandemic — R1/PR6

**DOI:** 10.1017/gmh.2024.83.pr6

**Affiliations:** Johns Hopkins University Bloomberg School of Public Health, United States

No accompanying comment.
